# Reliability and validity of Vietnamese version of the Knowledge, Attitudes, Access, and Confidence Evaluation questionnaire

**DOI:** 10.1038/s41405-025-00326-8

**Published:** 2025-03-29

**Authors:** Phi Ngoc Quang Tran, Vu Hoa Anh Dien, Truc Thi Hoang Nguyen, Kien Trung Nguyen, Nhi Thi Phuong Pham, Anh Lam Tu Nguyen, My Khanh Nguyen

**Affiliations:** Faculty of Dentistry, Van Lang University, Ho Chi Minh City, Vietnam

**Keywords:** Dental education, Continuing professional development in dentistry

## Abstract

**Objective:**

To effectively evaluate evidence-based dentistry (EBD) training outcomes, precise and validated assessment tools are essential. This study aimed to construct a Vietnamese version of the KACE questionnaire (Knowledge, Attitudes, Access, and Confidence Evaluation) and to assess its reliability and validity.

**Methods:**

After translating the original KACE questionnaire, we conducted tests for face validity, content validity, and discriminant validity test. Cronbach’s alpha and intraclass correlation coefficients (ICC) were calculated to evaluate the internal consistency and reliability of the Vietnamese KACE questionnaire.

**Results:**

Thirty lecturers and 280 dental students completed the KACE questionnaire. Cronbach’s alpha for the Attitude scale ranged from 0.90 to 0.97, for the Accessing Evidence scale from 0.90 to 0.96, and for the Confidence scale ranged from 0.83 to 0.93 across lecturers and dental student groups. The Knowledge scale had Cronbach’s alpha from 0.55 to 0.79. The overall ICC of 0.861 indicated that the measurement is stable and consistent.

**Conclusion:**

The Vietnamese adaptation of the KACE questionnaire is a reliable and valuable tool for assessing EBD competencies among Vietnamese dental students and practitioners.

## Introduction

Evidence-based dentistry (EBD), as defined by the American Dental Association (ADA) in 1999, is *“… an approach to oral health care that requires the judicious integration of systematic assessments of clinically relevant scientific evidence, relating to the patient’s oral and medical condition and history, with the dentist’s clinical expertise and the patient’s treatment needs and preferences* [1]. Over the past two decades, EBD has played a crucial role in enhancing the quality of dental treatment and promoting oral health outcomes. However, with the growing volume of publications, it is nearly impossible for clinicians to stay current with the advancements in dental interventions, clinical recommendations, and research that are relevant to their specific patients’ needs. This underscores the importance of equipping oral health care providers with the competencies essential for EBD, such as formulating answerable clinical questions, accessing and critically appraising evidence, integrating findings with patient needs, and applying the results in clinical practice [2].

Despite its significance, EBD is not yet widely integrated into daily practice of oral healthcare providers worldwide, particularly in low- and middle-income countries. A study by Minja et al. (2021) reported that knowledge and awareness of EBD among dentists in Nigeria and Rwanda were below average. No more than one-third of respondents could correctly identify definitions of key EBD-related terms, including evidence-based practice (30.7%), critical appraisal (31.6%), and systematic review (21.1%). Additionally, most dentists relied primarily on their clinical experience rather than practicing EBD to inform patient care decisions [3]. In Morocco, Kadri et al. (2022) reported that only 52.2% of 209 surveyed dentists were familiar with the concept of EBD, and just 34.9% reported frequent use of EBD in daily practice [4].

In light of these challenges, many dental institutions have begun integrating EBD into their undergraduate and postgraduate curricula [5–7]. To evaluate EBD training outcomes effectively, precise and validated assessment tools are essential. One such tool is the Knowledge, Attitudes, Access, and Confidence Evaluation (KACE) questionnaire. This questionnaire was introduced by Hendricson et al. in 2011 as a tool to assess the outcomes of EBD training in dental education [8]. Initially, its pilot version was developed based on several Evidence-Based Practice questionnaires designed for medical contexts [8–12]. Hendricson et al. adapted the items and response formats in the attitudes, confidence, and evidence-accessing sections from a medical to a dental context and developed a new knowledge scale that was more relevant to dentistry [8, 9]. Since its introduction, the KACE questionnaire has been widely used in various studies to evaluate EBD competencies and confidence in critical appraisal [3, 4, 13–15]. However, applying this questionnaire to evaluate EBD competencies in the Vietnamese dental professional community and among Vietnamese dental undergraduates requires a thorough linguistic and cultural adaptation. The primary objective of this study was to construct a Vietnamese version of the KACE questionnaire and assess its reliability and validity. Additionally, this study aimed to examine score differences between lectures and students before and after the EBD courses.

## Materials and methods

### Participants

The participants were 280 dental students and 30 lecturers at Van Lang University. The inclusion criteria were: (a) Fourth- and fifth-year dental students from the Faculty of Dentistry at Van Lang University who had completed courses on Evidence-Based Dentistry and Critical Thinking in Dental Practice, as well as lecturers from the same faculty; and (b) Participants who provided informed consent. The exclusion criteria was incompleted questionnaire responses.

### Study procedure

A cross-sectional descriptive study was conducted to determine the structural validity and internal consistency of the Vietnamese version, ensuring it is equivalent to the original version in terms of semantics, concepts, and content. There are different methods to adapt the questionnaire, including direct translation, back translation, committee assessment, and pilot studies.

#### Phase 1: Translating the questionnaire

The study utilized the KACE questionnaire, a self-administered instrument consisting of 35 items, originally developed by Hendricson [8]. These 35 questions were organized into four scales corresponding to the four dimensions of Evidence-Based Dental Practice.

An expert in dental education, proficient in English (IELTS 8.0), translated the questionnaire from English to Vietnamese. Subsequently, another expert with equivalent English proficiency (a lecturer at the English Language Institute, Van Lang University) translated it back into English. A native English speaker then reviewed the back-translated version for consistency with the original, ensuring both semantic and conceptual equivalence.

#### Phase 2: Content testing of the questionnaire development

A pilot group of 15 lecturers and students, who were not part of the main study, was randomly selected from the Faculty of Dentistry. These individuals reviewed all sections of the questionnaire, including both the questions and responses. They were then interviewed to assess the clarity and comprehensibility of the Vietnamese version, identifying any unclear or confusing terms. Based on their feedback, the researchers refined and adjusted the Vietnamese terms in the questionnaire to create a finalized version for the subsequent phases of the study.

#### Phase 3: Content validity test

A panel of five experts, including researchers, clinicians, and educators in the field of dentistry, reviewed the Vietnamese version of the questionnaire and provided detailed feedback. The panel offered suggestions regarding the content, language, and phrasing of each question. After incorporating these edits, the final version of the Vietnamese KACE questionnaire was approved for use in the main investigation.

#### Phase 4: Assessing internal consistency

Lecturers and students from the research sample were asked to complete the Vietnamese version of the KACE questionnaire. To evaluate internal consistency, Cronbach’s alpha coefficient was calculated.

#### Phase 5: Determining the reliability of the questionnaire

To assess reliability, 25% of the participants who had completed the Vietnamese version of the KACE questionnaire in the first round were randomly selected to complete it again two weeks later. This interval ensured that participants were unlikely to recall their previous responses. The Intraclass Correlation Coefficient (ICC) was then calculated to evaluate the consistency between the two sets of responses.

### Ethical consideration

This study was approved by the Research Ethics Committee for Biomedical Research at Van Lang University, with reference number 03/2024/HDD-IRB-VN01.078.

### Study measures

During the content testing phase of the questionnaire, data were collected from 15 volunteers, including their current position (student or lecturer), their rating of the comprehensibility of the Vietnamese version on a 10-point scale, and any words or phrases they found confusing or unclear in the Vietnamese version.

In the phases assessing structural validity, internal consistency, and reliability of the Vietnamese questionnaire, the data collected from participants included their gender, year of university graduation (for lecturers), academic year (for students), the number of correct answers in the Knowledge scale, agreement scores in the Attitudes scale, accessibility ratings in the Accessing Evidence scale, and confidence ratings in the Confidence scale. These scales were designed as 5-point Likert scales (1–5).

### Data analysis

Statistical analyses were performed using Stata software version 17.0 for Windows. All variables were tested for normality using the Shapiro-Wilk test (α > 0.05) and were summarized as mean scores and standard deviation (SD). The Chi-squared test was used to compare EBD knowledge, while the t-test was utilized to assess differences in the average scores of Attitudes, Access, and Confidence Evaluation between lecturers and the two student groups. The reliability of the KACE questionnaire was investigated using Cronbach’s alpha and corrected item-total correlations. Test-retest reliability was assessed using the ICC.

## Results

A total of 310 participants completed the Vietnamese version of the KACE questionnaire. Participants were 30 lecturers and 280 dental students from the Faculty of Dentistry, Van Lang University. There were no significant differences in gender distribution in both lecture and student groups.

**Fig. 1:**
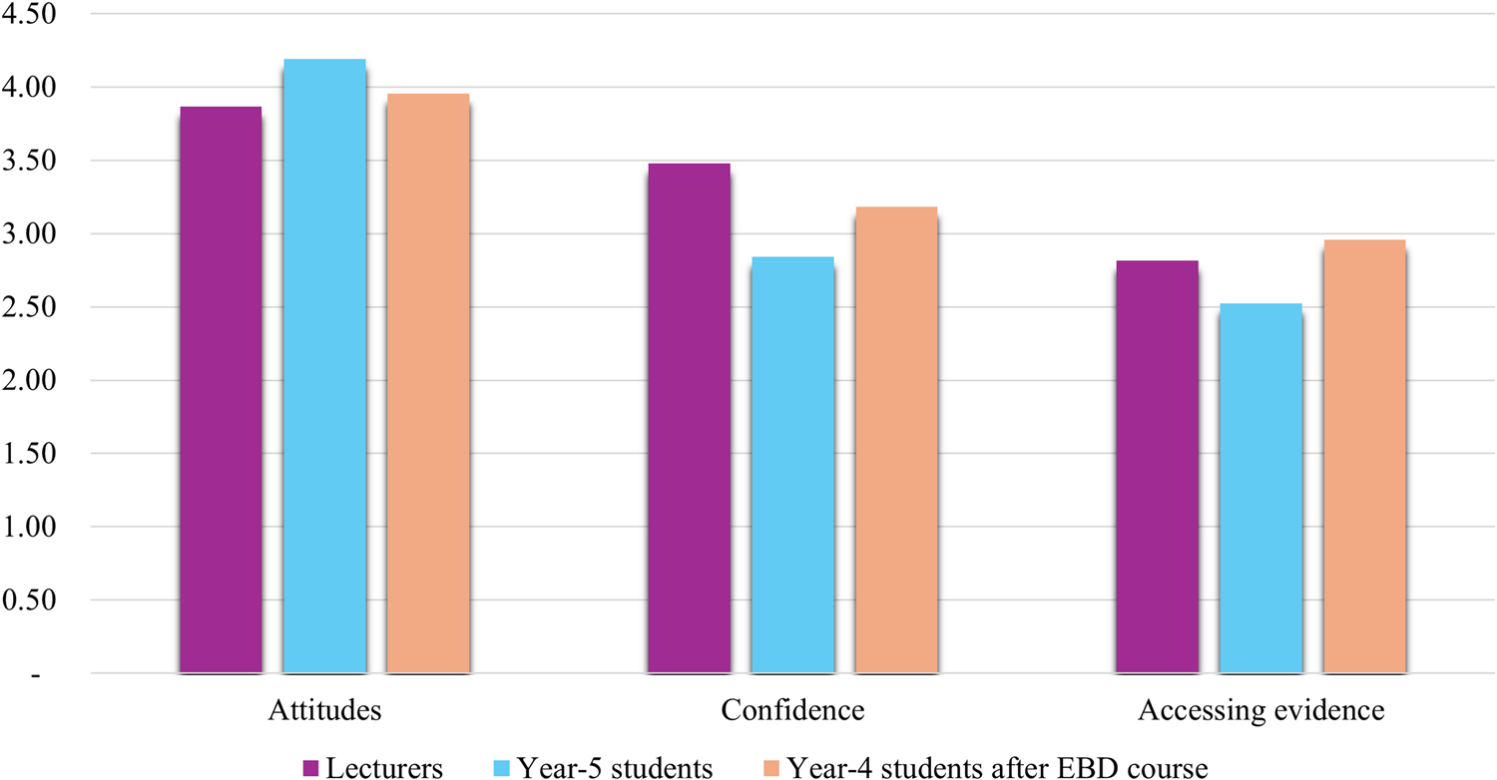
Comparison of Attitude, Confidence, and Access to Evidence scales between lecturers and students.

Average scores of KACE scale in each group of subjects were shown in Table 1. Knowledge was presented by a percentage of correct answers, while others were recorded by means and standard deviations. Lecturers had the highest scores compared to student groups. After taking the EBD course, however, the fourth-year student group significantly increased their scores. Figure 1 presents the average scores for Attitude, Confidence, and Accessing Evidence scales among lecturers, fifth-year students, and fourth-year students after the EBD course. No statistically significant differences were observed among the groups.

**Table 1:** Average scores of KACE scale in each group of participants.

Scores	Lecturers	Year-5 students	Year-4 students before course	Year-4 students after course
	(*n* = 30)	(*n* = 167)	(*n* = 113)	(*n* = 113)
Knowledge* (% correct)	52.0*	51.0	32.6	70.4*
**Attitudes (mean ± SD)**	3.87 ± 0.91	4.19 ± 0.73	3.55 ± 1.11	3.96 ± 0.82
**Confidence (mean ± SD)**	3.48 ± 0.86	2.84 ± 0.95	3.08 ± 0.99	3.18 ± 0.89
**Accessing evidence (mean ± SD)**	2.82 ± 0.79	2.52 ± 0.83	2.80 ± 0.96	2.96 ± 0.80

* Chi-squared test, *p*-value = 0.032.

### Validity

#### Face validity

A pilot test was conducted with 15 individuals who were not part of the main study. Participants were asked to complete the survey and provide feedback on any words, phrases, or sentences they found unclear or irrelevant. Some terms, such as “Critically Appraised Topics,” “sensitivity,” and “incidence,” were clarified for those who reported confusion. Most participants (97.1%) indicated that the questionnaire was clear and easy to understand.

#### Content validity

Content validity was determined by organizing a meeting of the expert panel as described in the Methods section. The expert panel members suggested changing the order of items, rephrasing, and explaining some scientific terms. The Vietnamese KACE questionnaire was then adjusted to a final version.

#### Discriminant validity

Discriminant validity was confirmed by the significant difference in scores between groups (Table 1). The fourth-year students who had not learnt EBD had lower scores than the fifth-year students who had already been trained in EBD. A significant difference was observed in the knowledge scores between lecturers and the fourth-year students after the EBD course (*p* = 0.032).

### Reliability

The Knowledge scale had a Cronbach’s alpha of 0.67. Overall, the Vietnamese KACE questionnaire showed good reliability, with the lowest Cronbach’s alpha observed for the Confidence scale (α = 0.90) and the highest for the Attitude scale (α = 0.96). Internal consistency for the Attitude scale ranged from 0.90 to 0.97, for the Accessing Evidence scale from 0.90 to 0.96, and for the Confidence scale ranged from 0.83 to 0.93 across lecturers and dental student groups (Table 2).

**Table 2:** Cronbach’s alpha of KACE questionnaire in each group of participants.

Cronbach’s alpha	Number of questions	Lecturers	Year-5 students	Year-4 students pre	Year-4 students post	Total
		(*n* = 30)	(*n* = 167)	(*n* = 113)	(*n* = 113)	(*n* = 310)
**Knowledge**	10	0.56	0.55	0.79	0.78	0.67
**Attitudes**	10	0.94	0.90	0.97	0.90	0.96
**Confidence**	9	0.83	0.88	0.93	0.91	0.90
**Accessing evidence**	6	0.90	0.90	0.96	0.95	0.93

Test-retest reliability was analyzed among 25% of participants for further assessment of reliability of Vietnamese KACE questionnaire. The correlations for each item in KACE scales were respectively reported as knowledge (0.665), attitudes (0.881), evidence accessing (0.891), and confidence (0.888). The overall ICC is 0.861.

## Discussion

The present study aimed to develop a Vietnamese version of the Knowledge, Attitudes, Access, and Confidence Evaluation (KACE) questionnaire and to assess its reliability and validity among dental students and lecturers at Van Lang University. Most participants demonstrated a positive attitude toward evidence-based dentistry (EBD), although a significant number had limited prior exposure to the concept. Adapting the KACE questionnaire is critical for evaluating evidence-based dental practice (EBP) in the Vietnamese context, as it provides valuable insights into the educational effectiveness of EBP curricula.

To establish validity, this study used multiple methods to assess both content and construct validity. A pilot test was first conducted with 15 individuals who were not participants of the main study to identify any unclear or irrelevant items in the questionnaire. Terms such as “Critically Appraised Topics”, “sensitivity”, and “incidence” were noted during this phase as requiring clarification. The positive feedback, with 97.1% of participants indicating that the questionnaire was clear and easy to understand, supports the instrument’s strong face validity. Ensuring clarity and accessibility is essential for obtaining high-quality responses and minimizing potential bias in the data collection process. Additionally, revisions based on expert feedback ensured that the final Vietnamese version of the KACE questionnaire comprehensively addressed key domains related to EBD. Ensuring strong content validity was important to guarantee the tool accurately reflected the intended concepts and was both relevant and suitable for the context. The successful adaptation of the KACE questionnaire provides a valuable framework for similar studies in Vietnam and emphasizes the importance of culturally adapted assessment tools in health education research. Although the English version of the questionnaire is widely used, the carefully translated, developed, and tested native-language KACE questionnaire will enhance its applicability and support broader research in EBD training [2, 16].

The Vietnamese version of the KACE questionnaire also demonstrated strong discriminant validity, as evidenced by significant differences in scores among groups with varying levels of EBD exposure. In our curriculum, the Evidence-Based Dentistry course is taught in the first semester of the fourth year, including one theory credit hour and one practice credit hour. It helps students develop knowledge and skills to identify clinical problems, search for and evaluate relevant studies, and apply evidence-based findings to diagnosis and treatment. By the end of the course, students are expected to assess the strength of evidence and practice searching for studies to support their clinical decisions. The Critical Thinking in Dental Practice course is taught in the second semester of the fourth year, including two theory credit hours and one practice credit hour. This course trains students to think critically based on evidence and foundational knowledge in their field. Surprisingly, our findings showed that lecturers’ knowledge scores were lower than those of fifth-year students. This distinction highlights the questionnaire’s capacity to differentiate between individuals with varying levels of knowledge and understanding. These findings are consistent with previous studies indicating that dental professionals and dental students often lack adequate knowledge of EBD principles [3, 13, 15]. Potential reasons for the discrepancies between their scores and those of students could be that some lecturers have never studied EBD, while others may have learned it but forgotten over time. Additionally, some may not fully recognize the importance of EBD. Through this study, we can provide evidence to propose faculty development strategies to the department leadership.

In educational research, Cronbach’s alpha is a widely used coefficient for assessing the reliability of a measurement instrument and the internal consistency of its items [2, 17]. Cronbach’s alpha for the knowledge scale was 0.67 overall, which is generally acceptable for this type of instrument. This is a reasonable result, as it represents a mix of groups with varying levels of knowledge, including lecturers and students who have completed different levels of education in this area. However, reliability varied across groups, with lower values among lecturers (α = 0.56) and fifth-year students (α = 0.55), but higher reliability in fourth-year students pre- and post-training (α = 0.79 and 0.78, respectively). These findings indicate that participants who recently completed structured EBD training tend to provide more consistent responses, likely because they share similar levels of understanding in relevant content. Similar patterns have been observed in studies where knowledge-based scales reflect varying baseline educational backgrounds [10, 11].

A possible explanation for the lower alpha value among lecturers (0.56) is that many may not have received formal training in EBD, resulting in greater variability in their responses. This confirms the importance of continuous professional development programs to address knowledge gaps and improve consistency in EBD-related competencies among educators.

Reliability was evaluated using the Intraclass Correlation Coefficient. The attitude scale exhibited the highest internal consistency (α = 0.96 overall), with reliability ranging from 0.90 to 0.97 across groups. This underscores the questionnaire’s effectiveness in consistently capturing attitudes toward EBD. Previous studies have reported similarly high reliability for attitude scales, reflecting consistent perspectives on the importance and relevance of EBD [10, 12, 13]. Test-retest reliability further supported the stability of the questionnaire, with Pearson correlations indicating strong agreement across scales.

Besides the main aim of this study, our results revealed an interesting finding that students showed a positive shift toward EBD after completing the course. Although not statistically significant, this change is still a noteworthy contribution to improving dental education in Vietnam. Similar findings have been reported in other studies. Asgari et al. observed this positive change and emphasized the role of educational interventions in shaping students’ attitudes toward EBD, encouraging its adoption in clinical practice [14]. Rodriguez-Fitzpatrick et al. also found that when students perceive EBD as engaging and valuable, they are more likely to apply it effectively in their future careers [18].

Traditionally, Vietnamese dental education has been very teacher-centered, where lecturers are seen as the main source of knowledge [19]. Because of this, both students and faculty may not be as accustomed to independently searching for and critically evaluating research. Additionally, the academic and clinical environment is often hierarchical, making it difficult for students or younger faculty to get evidence-based approaches [20]. Access to research is another challenge [21]. Since most quality studies are in English, those who are not fluent may struggle to use EBD resources effectively. Vietnamese dental education also emphasizes hands-on experience, so some clinicians may be hesitant to rely on published evidence, especially if it contradicts traditional practices. When adapting the KACE questionnaire, we considered these cultural factors to ensure relevance. We adjusted the wording to fit the local context and clarified key terms like “confidence in accessing evidence.” Feedback from Vietnamese dental educators and clinicians helped improve the questionnaire so it accurately reflects how EBD is understood and applied in Vietnam.

Our findings suggest that EBD education in Vietnamese dental schools needs to be more integrated and practical. Instead of limiting it to a single course, reinforcing EBD skills throughout the curriculum could help students retain and apply them better. Faculty training is also essential, as some lecturers may not have a strong background in EBD. Improving access to research, especially for non-English speakers, and using active learning methods like case discussions and journal clubs could make EBD more engaging. A more hands-on, continuous approach will better prepare students for evidence-based practice.

Potential confounding factors, such as prior research experience, academic performance, or learning styles, may influence EBD competencies. However, dental students in the same class share a similar background in research experience and academic performance, as they have not conducted research yet. Due to the limited number of lecturers, we did not divide them into multiple groups based on their research or teaching experience. Since the results are presented as mean values, this limitation is unlikely to have a significant impact. Integrating qualitative insights from participants would likely bring further useful insights.

This study has limitations. First, it used a convenience sample of students from a single dental university, limiting the generalizability of findings to the broader population of Vietnamese dental students. However, the impact of this selection bias is minimal, as the primary objective was to assess reliability and validity among students and lecturers at Van Lang University rather than provide a comprehensive picture of EBP across Vietnam. Second, evaluating the long-term impact of the EBD course would require re-testing fourth-year students simultaneously with fifth-year students after course completion. Future research should conduct a multi-institutional longitudinal study with more participants from across the country using the adapted KACE questionnaire. Cohort studies are also needed to evaluate the long-term impact of EBP education on daily dental practice and to provide a more comprehensive understanding of EBD competencies in Vietnamese dental education. An assessment component that measures students’ ability to apply EBD principles in clinical scenarios, such as case studies or simulated patient encounters, should be included in the next study.

## Conclusion

The Vietnamese adaptation of the KACE questionnaire is a reliable and valuable tool for assessing EBD competencies among Vietnamese dental students and practitioners. The findings of this study show that the developed course effectively enhances EBD knowledge and attitude in dental students. However, reinforcement through practical application is essential, and further studies are needed to evaluate the long-term impact of EBD education.

## Data Availability

The data supporting this article can be made available by the corresponding author upon request.
